# Evaluation of probiotic properties and complete genome analysis of lactic acid bacteria isolated from crested ibis *Nipponia nippon* feces

**DOI:** 10.3389/fmicb.2025.1552264

**Published:** 2025-04-09

**Authors:** Lei Yang, Jie Luo, Yan Zeng, Baoyue Zhang, Yang Wang, Gang Shu, Xiaoling Zhao, Juchun Lin, Haohuan Li, Funeng Xu, Wei Zhang, Hualin Fu, Felix Kwame Amevor, Rui Liu

**Affiliations:** ^1^Department of Basic Veterinary Medicine, Sichuan Agricultural University, Chengdu, China; ^2^Department of Animal Husbandry and Veterinary Medicine, Tongren Vocational and Technical College, Tongren, China; ^3^Animal Microecology Institute, College of Veterinary Medicine, Sichuan Agricultural University, Chengdu, China; ^4^Farm Animal Genetic Resources Exploration and Innovation Key Laboratory of Sichuan Province, Sichuan Agricultural University, Chengdu, Sichuan, China

**Keywords:** lactic acid bacteria, crested ibis, probiotic, antibacterial activity, the complete genome

## Abstract

**Introduction:**

Crested ibis (*Nipponia nippon*) is a rare bird whose intestinal tract is rich in lactic acid bacteria (LAB), but there is less research on LAB isolated from crested ibises.

**Methods:**

From the fecal samples, Twenty isolates were obtained from fecal samples and subjected to a series of tests, including biochemical identification, acid and bile tolerance assays, *in vitro* pathogen inhibition, cell surface hydrophobicity assessment, antibiotic susceptibility testing, and hemolytic activity evaluation to determine their probiotic potential. We fed *L. plantarum* E7 to mice to evaluate safety. Nanopore PromethION48 and the Illumina Novaseq sequencing platforms were used to sequence the genome of *L. plantarum* E7.

**Results:**

Five isolates (D1, D2, D6, E7 and D8) were able to survive under low acid and high bile salt conditions. Except for D8, the other four isolates (D1, D2, D6 and E7) exhibited inhibitory activity against tested pathogens. Strain E7 displayed the least resistance to antibiotics, and only E7 showed medium hydrophobicity. Further characterization identified strain E7 as *Lactiplantibacillus plantarum* (*L. plantarum*) through 16S rDNA sequencing. We did not observe adverse effects of *L. plantarum* E7 on growth performance, blood cell composition in mice. *L. plantarum* E7 consists of a circular chromosome and two circular plasmids. The chromosome encodes 3024 genes that associated with cell adhesion, acid and bile salt tolerance, antioxidant enzymes, as well as the production of secondary metabolites. In contrast, the plasmids contain fewer coding genes. Functional annotation via KEGG and GO database analysis indicated that the genes of *L. plantarum* E7 are primarily involved in carbohydrate metabolism, amino acid metabolism, vitamin and cofactor metabolism, biological process, and molecular function.

**Conclusion:**

This study provides a theoretical foundation for developing new probiotic for crested ibises.

## 1 Introduction

The crested ibis (*Nipponia nippon*), also known as the Oriental gem, is a vulnerable and endemic species that has attracted global conservation attention (Zhu et al., 2021). *Ex-situ* conservation efforts have significantly contributed in the recovery of the crested ibis populations in China, Japan, and South Korea. In captivity, adult crested ibises are typically housed in cages and fed a diet primarily consisting of loach (Zhu et al., 2021). However, living in confined spaces and consuming a single food source for extended periods may disrupt the composition and structure of the ibis' intestinal microbiota, potentially increasing the risk of intestinal diseases. Although antibiotics are commonly used to prevent and treat such diseases, the emergence of drug resistance underscores the need for alternative solutions (Lu et al., 2022). Probiotics and prebiotics, which are non-toxic and do not contribute to drug resistance, have emerged as promising alternatives (Ouwehand et al., 2016; Seddik et al., 2017; Abd El-Ghany et al., 2022).

LAB are among the most extensively studied probiotics. LAB ferment carbohydrates to produce lactic acid, colonize the oral and gastrointestinal tracts of animals, and play a critical role in maintaining gut microbiota balance (Akinyemi et al., 2024). Studies have shown that LAB can regulate intestinal microbiota (Zeng et al., 2023), promote the growth of beneficial bacteria, strengthen the intestinal epithelial barrier (Kumar et al., 2022), secrete antimicrobial substances (Sathiyaseelan et al., 2022) (such as bacteriocin, organic acid, hydrogen peroxide), and participate in immune responses (Shi et al., 2024). Moreover, Peng et al. (2022) found that *Lactobacillus rhamnosus* could resist *Salmonella* infections in chickens, presenting it as a promising alternative to antibiotics in farm animal management. LAB are widely available in various environments (Cheon et al., 2020; Haghshenas et al., 2023), with sources including animal intestines and feces, which have shown promising probiotic properties (Sirichokchatchawan et al., 2018). However, LAB isolated from the feces of the crested ibis remain underexplored.

Therefore, this study aimed to isolate lactic acid bacteria from the feces of crested ibises and evaluate their probiotic potential through a series of *in vitro* tests, including tolerance, antibacterial, drug sensitivity, and hydrophobicity tests, as well as *in vivo* safety evaluations. Pathogenic bacteria commonly found in the gastrointestinal tract of crested ibises were used to test the inhibitory properties of the isolated strains. In addition, we analyzed the complete genome sequences of the LAB strains to identify key functional genes. This study represents the first step toward identifying probiotics that could potentially prevent and treat intestinal diseases in crested ibises. The next step will focus on monitoring the effects of long-term LAB consumption on the intestinal microbiota and health of the crested ibis. Our findings not only contribute to improving the gut health of crested ibises but also provide valuable insights for microbiological intervention interventions in wildlife conservation.

## 2 Materials and methods

### 2.1 Bacterial strains and fecal sample collection

Four fecal samples were collected from four healthy adult crested ibises (two males and two females) housed at the Emei Mountain Crested Ibis Breeding Base in Leshan City, Sichuan Province, China (103.67°E, 29.37°N). Fresh feces were collected using sterile cotton swabs from the ibises' cages and transferred into sterile sampling tubes. The following bacterial strains were used for pathogen inhibition and hemolytic activity tests: *Escherichia coli* (*E. coli*) ATCC 25922, *Staphylococcus aureus* (*S. aureus*) ATCC 25923, *Enterotoxigenic Escherichia coli* (ETEC) CVCC 196, and *Salmonella typhimurium* (*S. typhimurium*) ATCC 14028, which were obtained from the pharmaceutical laboratory at Sichuan Agricultural University.

### 2.2 Isolation of LAB

Each fecal sample was homogenized in 9 mL of sterile saline, serially diluted, and plated onto MRS agar (Qingdao Haibo Biotechnology Co., Ltd). The plates were incubated at 37°C for 24–48 h under anaerobic conditions. Colonies exhibiting distinct morphologies were selected and purified through multiple re-streaking on MRS agar (Wang et al., 2023). Gram-positive pure isolates were subcultured in MRS broth (Qingdao Haibo Biotechnology Co., LTD) for further analysis. Biochemical identification of the isolates was performed by Hangzhou Microbial Reagent Co., Ltd. following *Bergey's Manual of Systematic Bacteriology* and *Isolation and Identification and Test Methods of Lactic Acid Bacteria*.

### 2.3 Acid and bile tolerance test

The 20 isolates were grown in MRS broth for 24 h, and the viable count was adjusted to 10^8^ CFU/mL. Acid tolerance was assessed by adjusting the pH of MRS broth to 1.0, 2.0, and 3.0 using 1 M HCl, while bile tolerance was evaluated by adding varying concentrations (0.3%, 1%, 2%) of bovine bile salts (Qingdao Haibo Biotechnology Co., LTD).

The isolates were incubated at 37°C, and viable counts were measured at 0 and 3 h for acid tolerance, and at 0 and 4 h for bile tolerance. The survival rate was calculated using the following formula below:


(1) $$\begin{array}{l} \text{Survival rate}\%\;=\left[\frac{\text{N}2}{\text{N}1}\right]*\;100 \end{array}$$

N1, the number of viable bacteria in MRS broth with pH 5.7 and 0.0% bile salt (CFU/mL); N2, the number of viable bacteria in MRS broth with different acidic conditions (pH 1.0, 2.0, and 3.0) and different bile salt concentrations (0.3%, 1%, and 2%) (CFU/mL).

### 2.4 *In vitro* inhibition of pathogenic bacteria test

To evaluate the antibacterial activity of the isolates, the antibacterial activity of the isolates was evaluated using the agar well diffusion method against the pathogens *E. coli* ATCC 25922, *S. aureus* ATCC 25923, and *S. typhimurium* ATCC 14028. Both bacterial suspensions, whole bacterial broth and cell-free supernatants (CFS) were tested separately for inhibitory activity (Hu et al., 2023).

### 2.5 Antibiotic sensitivity tests

Antibiotic susceptibility was evaluated using the disk diffusion method, following Clinical and Laboratory Standards Institute (CLSI) guidelines. Isolates and an antibiotic-impregnated paper disk (Hangzhou Microbial Reagent Co., Ltd.) were placed on MRS agar plates. After 24 h of incubation at 37°C, the experimental results were compared with CLSI M100-S17 (2007) (Jorgensen and Hindler, 2007) standard to determine the antibiotic sensitivity (Akinyemi et al., 2024). Antibiotics tested included penicillin (10U), cefoperazone (75 μg), cefradine (30 μg), carbenicillin (100 μg), chloramphenicol (30 μg), tetracycline (30 μg), doxycycline (30 μg), erythromycin (15 μg), ciprofloxacin (5 μg), gentamicin (10 μg), kanamycin (30 μg), and vancomycin (30 μg).

### 2.6 LAB cell surface hydrophobicity assay

The hydrophobicity of LAB cell surfaces was assessed using the hydrocarbon affinity method (Chen et al., 2020). The isolates were inoculated in MRS broth for 24 h. The culture was centrifuged (12,000 × g, 4°C, 10 min) to harvest the cells and washed 2–3 times by PBS (pH 7.4) buffer, and then resuspended in PBS to adjust concentration (OD_600nm_ = 0.26–0.3). A 3 mL bacterial suspension was mixed with 1 mL xylene by vortexing for 5 min, and the OD_600nm_ was measured after 30 min. and three independent tests were repeated. The degree of cell surface hydrophobicity was classified as low (0%−29%), medium (30%−59%), or high (60%−100%) (Niederle et al., 2019). The hydrophobicity was calculated using the following formula:


(2) $$\begin{array}{l} \text{Hydrophbicity}\%=\left[\frac{\text{A}0-\text{A}}{\text{A}0}\right]*\;100 \end{array}$$

A0, absorbance value of LAB before xylene treatment; A, absorbance value of LAB after xylene treatment.

### 2.7 Hemolytic activity

Hemolytic activity was assessed by streaking fresh bacterial cultures onto blood agar plates containing 5%−10% sheep blood and incubating at 37°C for 24 h. ETEC CVCC 196 served as a positive control.

### 2.8 Characterization of antibacterial substances in strain E7

The antimicrobial activity of strain E7 was evaluated using the agar well diffusion method. The CFS of strain E7 were collected and divided into four treatments: the first group was heat-treated (boiled) for 10 min, the second group was neutralized to pH 7 with 6 N NaOH, the third group was treated with 0.5 mg/mL catalase and the fourth group was left untreated. After 12 h at 37°C, the diameter of the circle of inhibition was measured and recorded. Finally, Minimum Inhibitory Concentration (MIC) was determined using the microdilution broth method.

### 2.9 Growth curve

The activated bacterial solution was inoculated into MRS broth at 1% (v/v) (volume of solute/volume of solution), incubating at 37°C. The OD_600nm_ was measured every 2 h for 24 h (Cao et al., 2024).

### 2.10 Molecular identification

The isolates were cultured at 4 mL MRS broth at 37°C overnight and the culture was centrifuged (12,000 × g, 4°C, 10 min) to harvest the cells and washed 2–3 times by sterile saline. Genomic DNA was extracted using a Rapid Bacterial Genomic DNA Isolation Kit (Sangon Biotech, China). The primers 27F and 1492R were used for PCR amplification, with an expected amplicon size of 1,500 bp and the DNA fragments were sequenced (Tsingke biotechnology). The PCR products were sequenced and analyzed using BLAST against the GenBank database. Species identification required >99% sequence identity.

### 2.11 *In vivo* safety assessment

Four-week-old male Kunming mice (20.0 ± 0.5 g) were obtained from Da Shuo Biotechnology Co., Ltd., Chengdu, China and housed in controlled conditions with free access to antibiotic-free food and water. The animal study was reviewed and approved by the Institutional Animal Care and Use Committee of Sichuan Agricultural University (approval number: DY2022203014).

The mice were pre-fed for 7 days in a well-ventilated environment with a normal light-dark cycle and good illumination (temperature 22 ± 1.5°C; free access to water). Subsequently, 30 mice were randomly and equally divided into three groups: NC Group (Negative control group, fed with 0.2 mL of saline alone with the standard diet), BL Group (low-dose group, fed with 0.2 mL of 4.0 × 10^8^ CFU/mL of *L. plantarum* E7 solution), and BH Group (high-dose group, fed with 0.2 mL of 4.0 × 10^10^ CFU/mL of *L. plantarum* E7 solution). Each group was housed in two cages with five mice per cage. Each mouse was fed 3–5 g of diet per day. The health status, feed intake, and fecal condition were recorded daily.

The experimental period lasted 14 days. The mice were weighed every 2 days. The number of deaths, average daily weight gain (ADG) of mice were recorded and measured.

On day 15, the whole blood and serum of mice were collected, and then the mice were euthanized. The liver and spleen were taken to measure organ indices. Thereafter, the whole blood samples were used to detect hematological parameters by an Automatic Blood Cell Analyzer (Shenzhen Jing Xintai Electronic Equipment CO., Ltd, Shenzhen, China).

### 2.12 Genome sequencing, assembly, and annotation

Genomic DNA was extracted using the cetyltrimethylammonium bromide (CTAB) method. The genome sequencing was then performed by Personal Biotechnology Company (Shanghai, China) by using the Nanopore PromethION48 and the Illumina Novaseq. Flye and Unicycler software (Syrokou et al., 2022) was used to assemble the data obtained by Nanopore platform sequencing. Subsequently, all assembled results were integrated to generate a complete sequence. Finally, the genome sequence was acquired after rectification by using pilon software. CGview (Stothard and Wishart, 2005) was used to give an overview of the genome information, while the genome sequences were uploaded to the NCBI database with an accession number of CP158575. The Carbohydrate-Active enzymes (CAZy) database (Lombard et al., 2014) was used to predict Carbohydrate-Active enzymes. Functional annotation was performed using the Kyoto Encyclopedia of Genes and Genomes (KEGG) and the Gene Ontology (GO) (Moriya et al., 2007) databases. AntiSMASH was used to search for secondary metabolites. The Virulence Factors of Pathogenic Bacteria (VFDB) database and the Comprehensive Antibiotic Resistance (CARD) database (Chen et al., 2016) were used to retrieve the pathogenicity and antibiotic resistance genes.

### 2.13 Statistical analysis

Data are presented as mean ± standard deviation and analyzed using SPSS version 27.0. The difference was evaluated by one-way ANOVA with Duncan's test. Statistical significance was set at *P* < 0.05.

## 3 Result

### 3.1 Morphological and phenotypic characteristics

A total of 20 isolates were obtained from crested ibis fecal samples (Table 1), exhibited characteristic small, pinpoint, creamy-white colonies with spherical and rod-shaped morphology. Gram-positive nature, catalase, hippuric acid, and gelatin liquefaction negativity, and both coccus and rod-shaped morphology.

**Table 1:** Morphological, physiological, and biochemical characteristics of lactic acid bacterial strains isolated from the fecal samples of crested ibis.

**Characteristic**	**1**	**2**
Isolates	D1, D2, D3, D4, D5, D6, D7, D8, D9, D11, D12, D13, D14, D15, D16, D17, D18, D19, D20	E7
Morphology	Coccus	Rod
Gram reaction	+	+
Catalase	-	-
Gelatin liquefaction	-	-
Sucrose	6/19	+
Xylose	17/19	+
Salicin	+	+
Glucose	+	+
Lactose	9/19	+
Inulin	16/19	+
Raffinose	-	+
Cellobiose	+	+
Esculin	+	+
Maltose	10/19	+
Sorbitol	5/19	+
Mannitol	7/19	+
Hippuric acid	-	-

+, positive or weakly positive reaction; –, negative reaction. Number, the number of positive reactions.

### 3.2 Acid tolerance

The tolerance test results for 20 LAB isolates at different pH levels are presented in Table 2. At pH 3.0, most isolates demonstrated high tolerance (*P* < 0.05). At pH 2.0, all isolates except strain D8 maintained viable bacterial counts above 10^4^ CFU/mL (*P* < 0.05). At pH 1.0, only strains D1, D6, E7, and D20 had viable counts exceeding 10^3^ CFU/mL, while no viable bacteria were detected in the remaining strains.

**Table 2:** Acid resistance capacity of LAB isolates under different acid conditions.

**Strains**	**pH 3**	**pH 2**	**pH 1**
D1	76.14 ± 0.57^def^	57.20 ± 2.68^cde^	38.10 ± 6.14
D2	81.82 ± 1.21^ab^	53.33 ± 1.57^def^	–
D3	72.75 ± 2.32^f^	52.46 ± 1.07^def^	–
D4	75.82 ± 3.47^ef^	49.01 ± 0.55^f^	–
D5	65.87 ± 1.29^g^	52.91 ± 3.38^def^	–
D6	77.80 ± 0.27^cde^	68.52 ± 1.06^a^	37.74 ± 3.99
E7	74.91 ± 0.76^ef^	61.09 ± 4.19^bc^	36.36 ± 4.18
D8	78.49 ± 0.84^bcde^	–	–
D9	79.53 ± 0.61^bcd^	57.48 ± 3.07^cd^	–
D10	65.83 ± 1.21^g^	51.47 ± 2.91^ef^	–
D11	74.78 ± 3.35^ef^	65.23 ± 1.86^ab^	–
D12	65.88 ± 2.73^g^	56.39 ± 0.70^cde^	–
D13	77.40 ± 0.95^cde^	59.59 ± 6.13^c^	–
D14	79.64 ± 0.07^bcd^	59.47 ± 4.48^c^	–
D15	84.18 ± 0.95^a^	61.85 ± 4.24^bc^	–
D16	74.90 ± 3.84^ef^	51.52 ± 1.11^ef^	–
D17	74.97 ± 0.89^ef^	49.09 ± 2.93^f^	–
D18	75.86 ± 1.59^ef^	52.63 ± 3.03^def^	–
D19	64.43 ± 3.31^g^	51.68 ± 0.97^def^	–
D20	80.49 ± 0.12^bc^	61.95 ± 4.14^bc^	35.23 ± 3.52

–, No viable bacteria were detected. The different letters represent significant diverse and the same letters represent no significant diverse in one queue. The three values for each sample are expressed as mean ± SD.

### 3.3 Bile tolerance

The tolerance results of 20 LAB isolates under different concentrations of bile salt are presented in Table 3. All strains showed high tolerance at 0.3% bile salt, with viable bacterial counts above 10^6^ CFU/mL (*P* < 0.05). At 1% bile salt, all strains except D3, D4, D12, D17, and D18 maintained counts above 10^4^ CFU/mL (*P* < 0.05). At 2% bile salt, only isolates D1, D2, D6, E7, and D8 had viable counts above 10^2^ CFU/mL, with no viable bacteria detected in other strains (*P* < 0.05). Strain E7 had a particularly high viable count exceeding 10^5^ CFU/mL (*P* < 0.05). Among the 20 strains, only five demonstrated good tolerance to low pH and high bile salts, warranting further analysis.

**Table 3:** Survivability of the LAB isolates under different concentrations of bile salt.

**Strains**	**0.3%**	**1%**	**2%**
D1	72.56 ± 4.68^bcd^	61.37 ± 0.67^bcd^	41.85 ± 6.41^b^
D2	69.06 ± 3.20^d^	57.98 ± 2.89^de^	26.01 ± 8.51^c^
D3	74.39 ± 3.91^abcd^	–	–
D4	75.27 ± 4.59^abc^	–	–
D5	79.28 ± 1.32^a^	67.12 ± 4.88^a^	–
D6	80.13 ± 1.83^a^	58.75 ± 4.96^cde^	28.53 ± 2.95^c^
E7	78.87 ± 3.71^ab^	60.46 ± 3.13^bcd^	59.64 ± 1.36^a^
D8	74.19 ± 5.23^abcd^	65.80 ± 0.43^ab^	46.63 ± 2.74^b^
D9	80.00 ± 2.75^a^	65.25 ± 2.54^ab^	–
D10	76.15 ± 3.12^ab^	54.36 ± 0.24^ef^	–
D11	75.75 ± 1.04^abc^	67.63 ± 3.39^a^	–
D12	74.75 ± 4.16^abcd^	–	–
D13	78.15 ± 2.04^ab^	50.86 ± 4.03^f^	–
D14	76.86 ± 1.17^ab^	58.18 ± 2.05^de^	–
D15	79.82 ± 2.74^a^	54.59 ± 2.65^ef^	–
D16	75.65 ± 3.51^abc^	67.56 ± 1.72^a^	–
D17	69.86 ± 0.88^cd^	–	–
D18	75.61 ± 2.64^abc^	–	–
D19	78.60 ± 2.42^ab^	63.64 ± 2.62^abc^	–
D20	75.18 ± 3.83^abc^	54.55 ± 2.11^ef^	–

–, No viable bacteria were detected. The different letters represent significant diverse and the same letters represent no significant diverse in one queue. The three values for each sample are expressed as mean ± SD.

### 3.4 Antimicrobial activity

**Figure 1:**
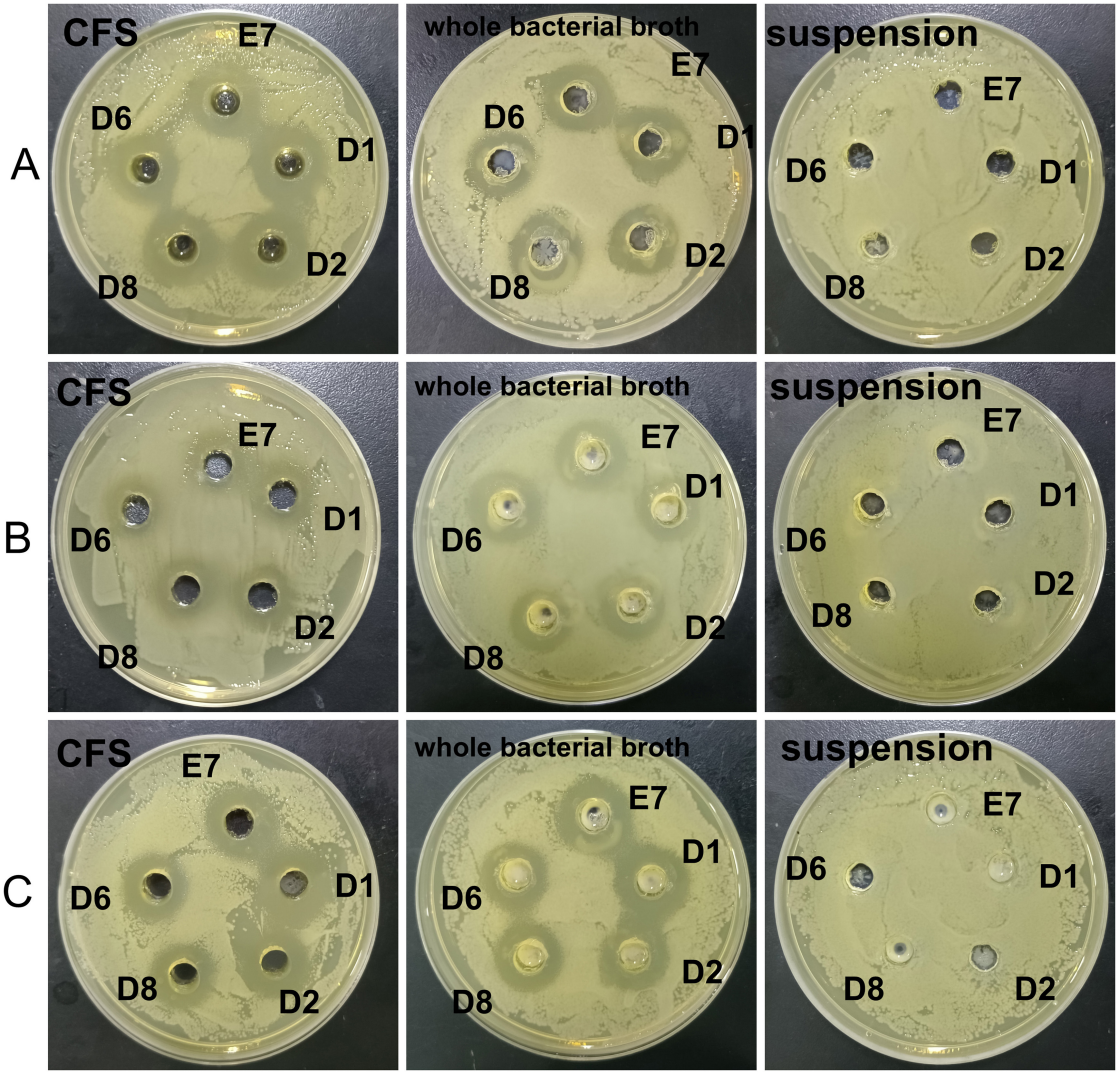
Antimicrobial activity of 5 LAB isolates. **(A)** Effects of isolates against *E. coli* ATCC 25922. **(B)** Effects of isolates against *S. typhimurium* ATCC 14028. **(C)** Effects of isolates against *S. aureus* ATCC 25923.

Five LAB isolates exhibited varying degrees of antibacterial activity against three pathogens (*E. coli, S. aureus*, and *S. typhimurium*) as shown in Figure 1. CFS and whole bacterial broths were more effective in inhibiting pathogens, and the bacterial proteins had no inhibitory capacity. CFS was selected to continue the study and the diameter of the circle of inhibition of CFS of the isolates was measured. All isolates, except D8 (Table 4), showed different inhibition zones (>14 mm) against the pathogens. Strain D8 showed significantly lower inhibition against *S. typhimurium* compared to other strains (*P* < 0.05).

**Table 4:** Antibacterial activities of CFS of LAB isolates.

**Strains**	***E. coli*** **ATCC 25922**		***S. typhimurium*** **ATCC 14028**		***S. aureus*** **ATCC 25923**	
D1	+	14.31 ± 0.21	++	19.83 ± 3.10	++	16.59 ± 0.76^a^
D2	+	14.43 ± 2.08	+	15.43 ± 3.68	++	18.13 ± 2.13^a^
D6	++	16.17 ± 0.60	++	17.36 ± 3.92	++	16.58 ± 3.07^a^
E7	++	16.53 ± 0.86	++	16.59 ± 1.57	++	16.65 ± 1.28^a^
D8	++	16.35 ± 2.17	+++	15.60 ± 2.01	+	12.04 ± 1.19^b^

The different letters represent significant diversity and the same letters represent no significant diversity in one queue. The three values for each sample are expressed as mean ± SD.

±, 8 mm < < zone diameters ≤ 12 mm; +, 12 mm < < zone diameters ≤ 16 mm; ++, 16 mm < < zone diameters ≤ 20 mm; +++, 20 mm < < zone diameters.

### 3.5 Antibiotic susceptibility

Selected isolates exhibited multi-drug resistance in Table 5. All strains were sensitive to penicillin and resistant to vancomycin. Strain D1 showed resistance to eight different antibiotics, while strains D2, D6, and D8 showed resistance to four different antibiotics. Strain E7 was resistant to cefradine, ciprofloxacin, and vancomycin. Although LAB are generally considered safe, the possibility of gene transfer in the gut of the crested ibis suggests selecting strains with less resistance. Strain E7 was found to be safer than the others.

**Table 5:** Susceptibility to antimicrobial agent of LAB isolates.

	**Antibiotics**											
**Strains**	**PNC**	**CFP**	**CE**	**CB**	**CHL**	**TE**	**DC**	**ERY**	**CIP**	**GM**	**KAN**	**VAN**
ATCC25923	S	S	S	S	S	S	S	S	S	S	S	S
D1	S	M	R	R	S	R	R	M	R	R	R	R
D2	S	M	R	S	S	M	S	M	R	S	R	R
D6	S	S	M	R	S	R	S	M	S	R	S	R
E7	S	S	R	S	M	S	S	M	R	S	M	R
D8	S	S	R	S	M	R	S	S	R	S	M	R

PNC, penicillin; CFP, cefoperazone; CE, cephradine; CB, carbenicillin; CHL, chloramphenicol; TE, tetracycline; DC, doxycycline; ERY, erythromycin; CIP, ciprofloxacin; GM, gentamicin; KAN, kanamycin; VAN, vancomycin.

R, Resistance; M, medium sensitivity; S, Sensitive.

### 3.6 Cell surface hydrophobicity

**Figure 2:**
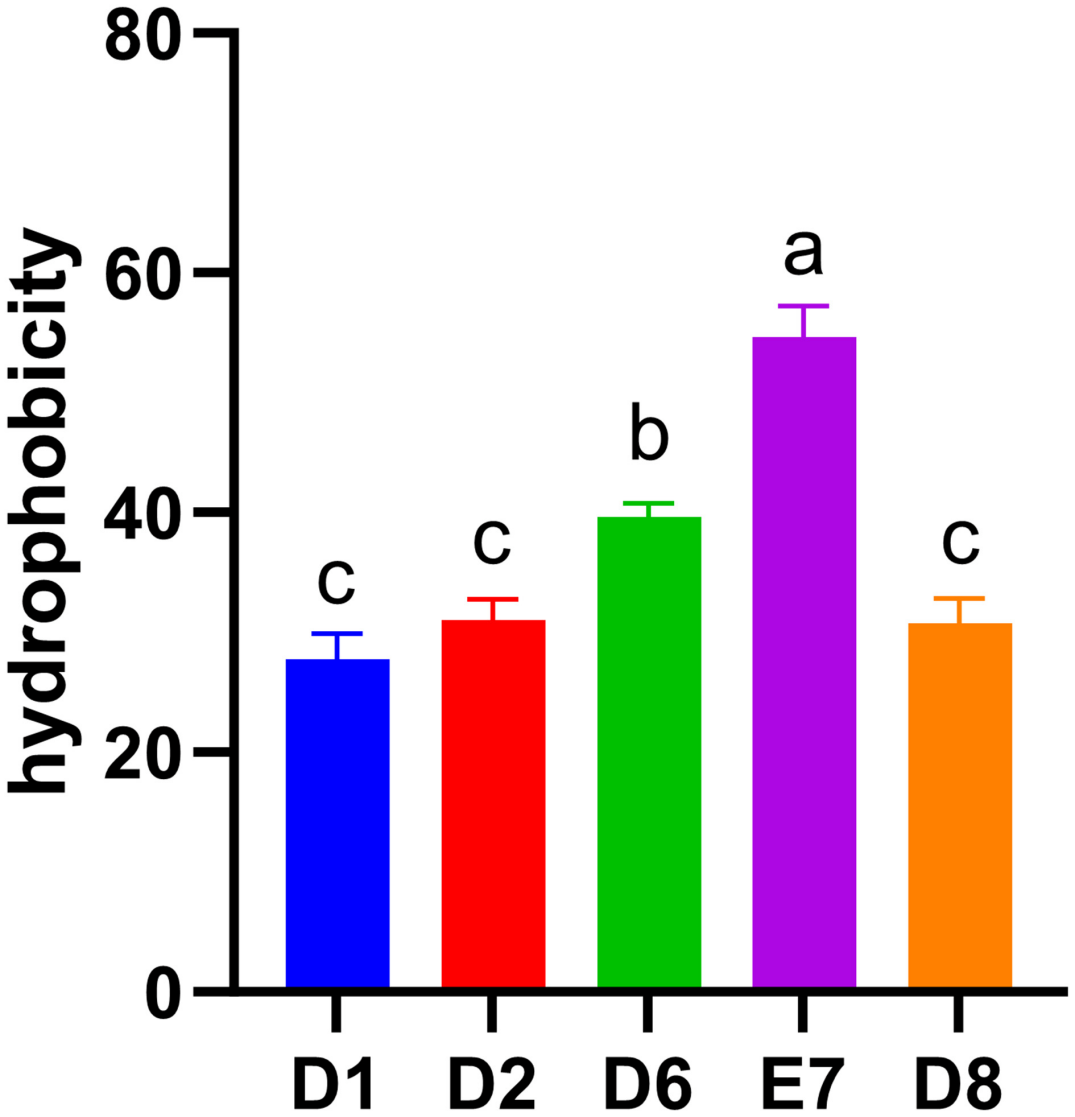
Hydrophobicity of five LAB isolates to xylene. Different letters represent significant difference (*P* < 0.05).

The cell surface hydrophobicity of LAB, a measure of adhesion to intestinal epithelial cells, varied among the five isolates (Figure 2). Strain E7 and D6 exhibited significant medium hydrophobicity (54.62%, 39.66%) (*P* < 0.05), while strain D2 and D8 also showed medium hydrophobicity (31.04%, 30.74%). Strain D1 exhibited low hydrophobicity (27.80%). Among the five isolates, strain E7 had the highest hydrophobicity (*P* < 0.05) and was selected for subsequent studies.

### 3.7 Hemolytic activity

**Figure 3:**
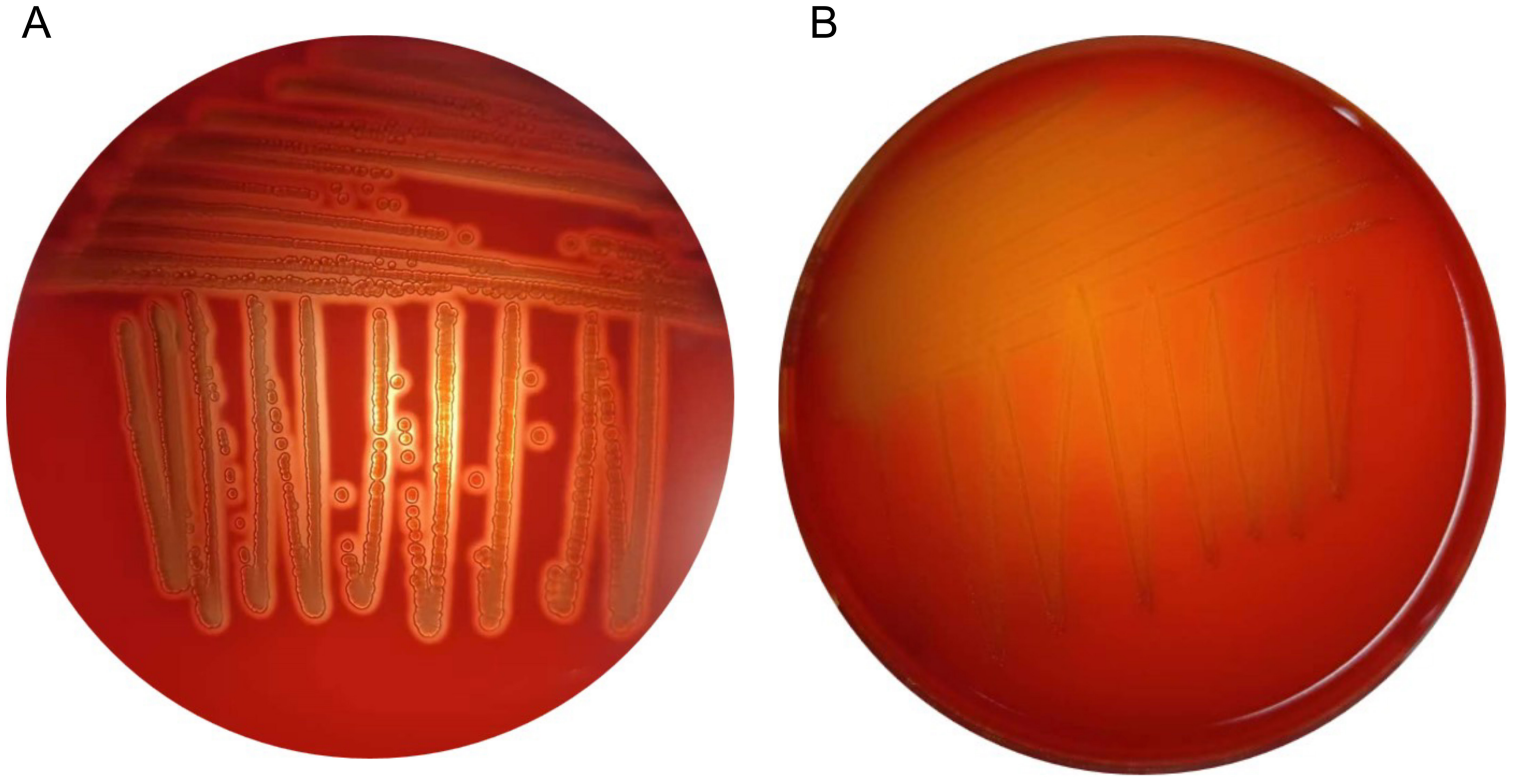
**(A)** Hemolysis results of ETEC CVCC196. **(B)** Hemolysis results of strain E7.

In hemolytic activity tests, ETEC CVCC 196 exhibited β-hemolysis (Figure 3A), whereas strain E7 showed no hemolytic activity (Figure 3B).

### 3.8 Characterization of antibacterial substances in strain E7

**Figure 4:**
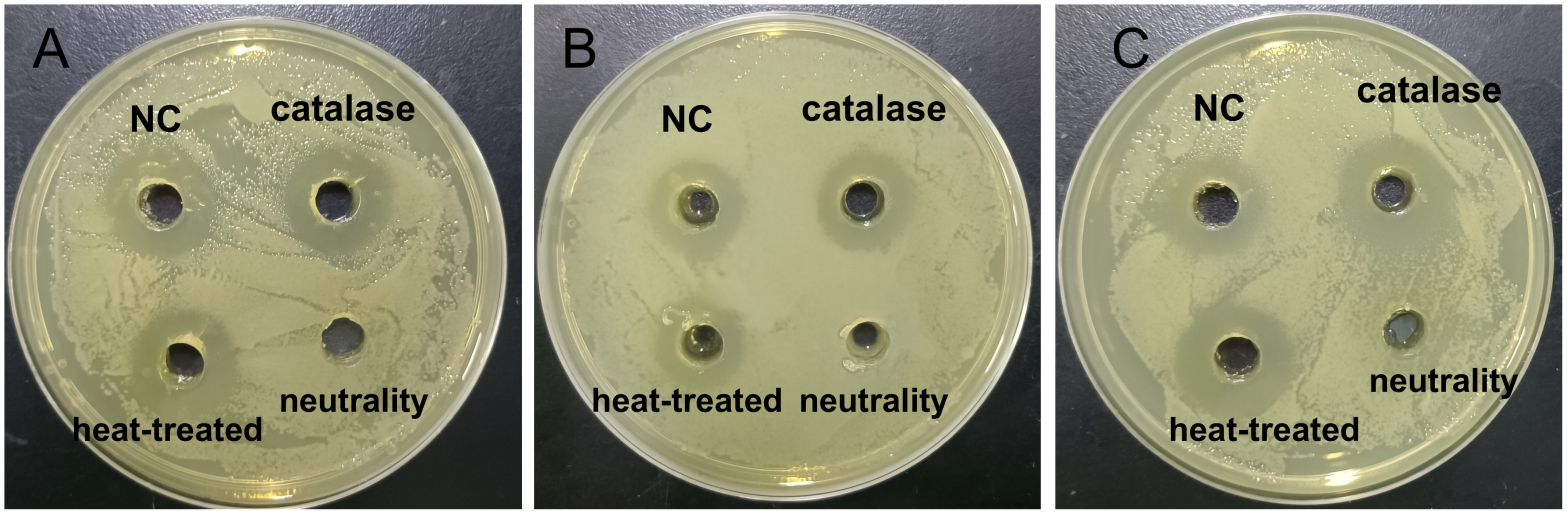
Results of antimicrobial substances in CFS of strain E7. **(A)** Effects of strain E7 against *E. coli* ATCC 25922. **(B)** Effects of strain E7 against *S. typhimurium* ATCC 14028. **(C)** Effects of strain E7 against *S. aureus* ATCC 25923.

**Figure 5:**
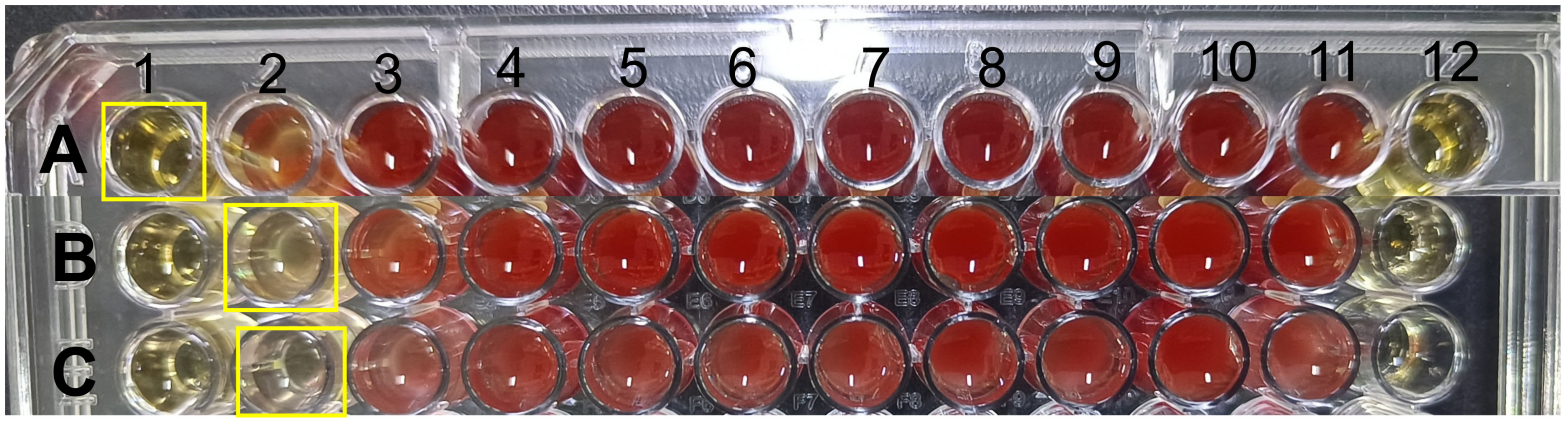
Results of MIC of strain E7. **(A)** MIC of CFS of strain E7 against *E. coli* ATCC 25922. **(B)** MIC of CFS of strain E7 against *S. typhimurium* ATCC 14028. **(C)** MIC of CFS of strain E7 against *S. aureus* ATCC 25923. The concentration of CFS in the first well was one-fourth, the concentration of CFS in the second well was one-eighth, and it was diluted sequentially to the 10th well. The 11th well is a positive control with bacterial solution and the 12th well is a negative control with CFS.

The antimicrobial properties of strain E7 were further characterized (Figure 4). The antimicrobial substances in the CFS of strain E7 were heat-resistant. When the CFS was neutralized to pH 7, the bacteriostatic activity was lost, suggesting that organic acids might be responsible for its antimicrobial effect. The Minimum Inhibitory Concentration (MIC) of CFS from strain E7 against *E. coli, S. aureus*, and *S. typhimurium* was 1/4, 1/8, and 1/8, respectively (Figure 5).

### 3.9 Growth curve

**Figure 6:**
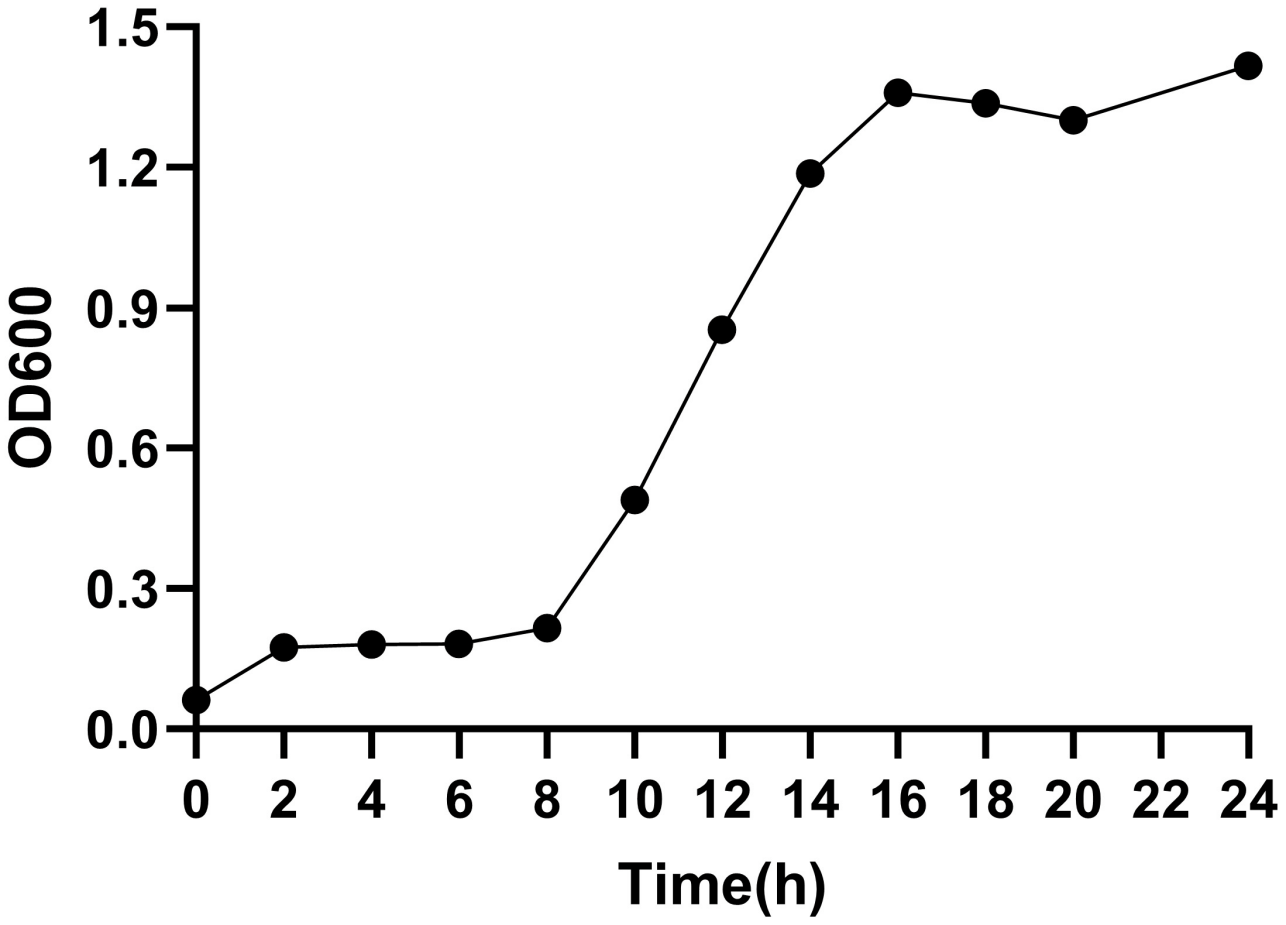
Growth curve of strain E7.

Figure 6 shows the growth curve of strain E7, which entered the logarithmic phase at 8 h and reached the stationary phase at 16 h, with a maximum OD600nm of 1.42.

### 3.10 Molecular identification of LAB strains

**Figure 7:**
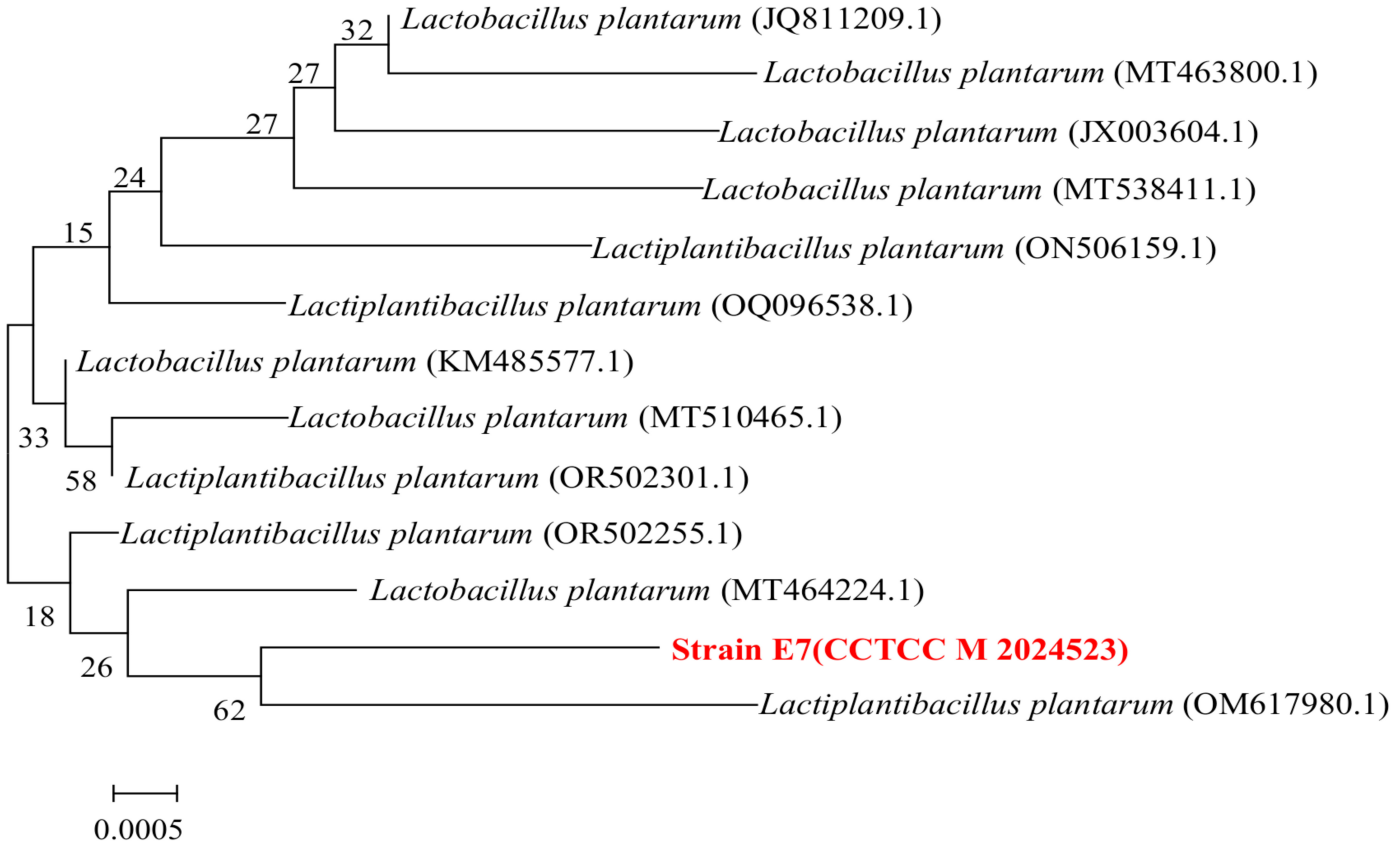
Phylogenetic relationships of strain E7 based on 16S rDNA gene sequences.

Strain E7 was further identified by 16S rDNA sequencing and phylogenetic analysis, showing 99% sequence similarity to *Lactiplantibacillus plantarum* based on BLASTn. The sequence of the strain E7 was uploaded to the China Center for Type Culture Collection (CCTCC M 2024523), and the phylogenetic tree was constructed using MEGA 11.0 (Figure 7). The branch tree is constructed using Neighbor-Joining method and evaluated Bootstrap values (Bootstrap > 1,000).

### 3.11 Growth performance of mice

**Figure 8:**
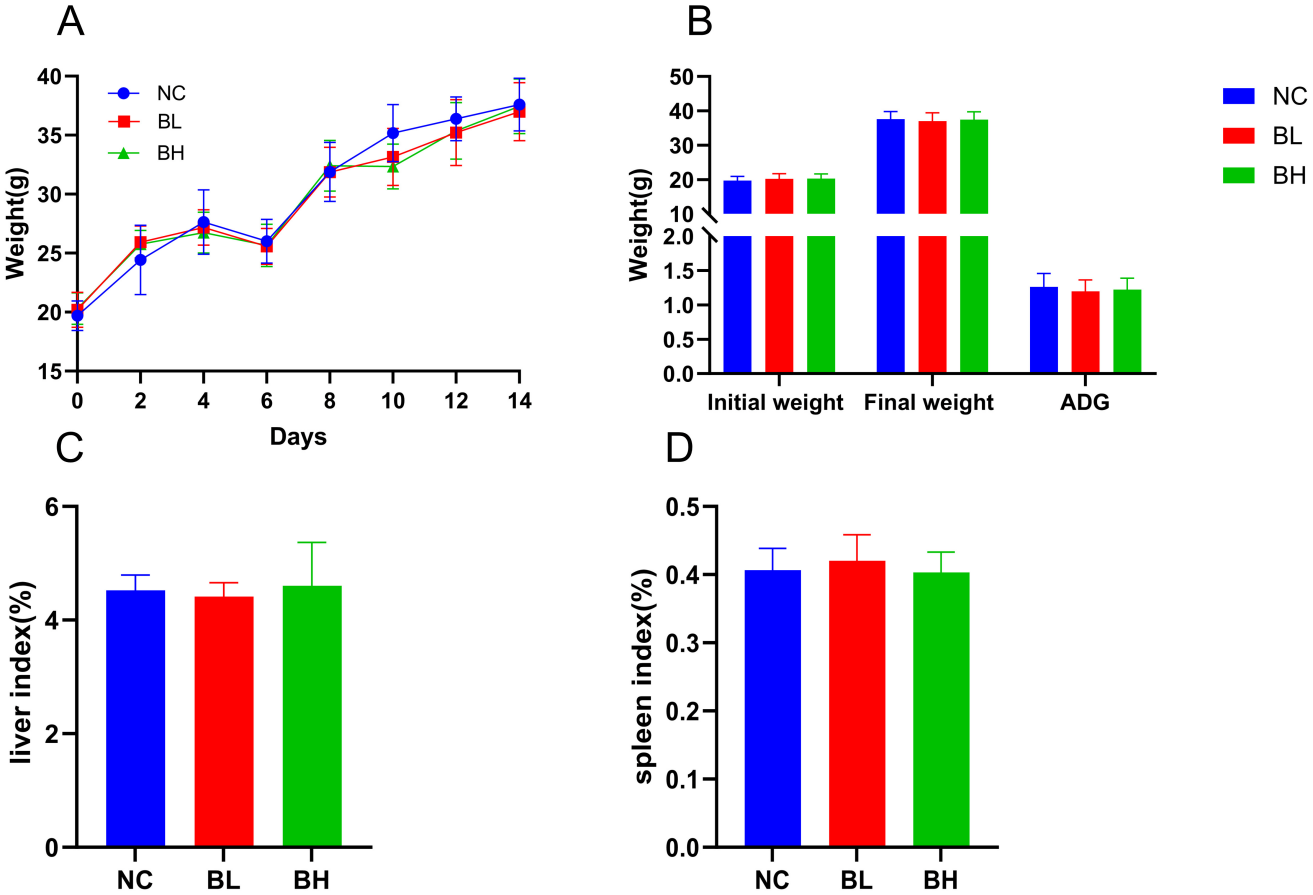
Growth indicators of *L. plantarum* E7 *in vivo* safety evaluation in mice. **(A)** Weight changes of mice. **(B)** Initial body weight (g), final body weight (g), and ADG (g) of mice. **(C)** Liver index of mice. **(D)** Spleen index of mice.

Ducircular experimental period, all of the mice were healthy, none of them died, and there was no loss of appetite or lethargy. Analysis of body weight, final body weight, and ADG showed that *L. plantarum* E7 supplementation had no negative effect on growth performance (Figures 8A, B). No significant differences were found in organ indices (liver, spleen) between the NC, BL, and BH groups (*P* > 0.05) (Figures 8C, D).

### 3.12 Analysis of hematological parameters

Hematological parameters are shown in Table 6. Except for platelet counts (PLT), there were no significant differences between BL and BH groups compared to the NC group (*P* > 0.05). The PLT in BL group and BH group were significantly higher than the NC group (*P* < 0.05).

**Table 6:** The hematological parameters of each group of mice.

**Parameters**	**NC group**	**BL group**	**BH group**	**Parameters**	**NC group**	**BL group**	**BH group**
WBC, 10^9^/L	4.18 ± 1.29	4.93 ± 1.00	5.05 ± 0.85	HGB, g/L	149.33 ± 15.16	150.83 ± 6.4	147.60 ± 8.14
Lymph, 10^9^/L	3.33 ± 1.26	4.08 ± 0.64	4.32 ± 0.96	HCT, %	49.21 ± 4.88	48.81 ± 2.15	48.18 ± 3.13
Mon, 10^9^/L	0.12 ± 0.04	0.15 ± 0.05	0.14 ± 0.1	MCV, fL	50.18 ± 1.27	48.75 ± 1.56	49.62 ± 0.59
Gran, 10^9^/L	0.73 ± 0.27	0.80 ± 0.20	1.08 ± 0.45	MCH, pg	15.15 ± 0.36	15.05 ± 0.50	15.14 ± 0.32
Lymph,%	78.9 ± 4.62	81.65 ± 2.99	77.36 ± 3.37	MCHC, g/L	303.83 ± 4.70	305.17 ± 2.14	306 ± 4.36
Mon, %	3.05 ± 0.80	3.02 ± 0.34	3.23 ± 0.77	RDW, %	14.42 ± 0.30	14.5 ± 0.81	14.9 ± 0.45
PDW, %	16.25 ± 0.3	16.43 ± 0.2	16.58 ± 0.16	PLT, 10^9^/L	1613 ± 126.18^a^	1819.66 ± 149.37^b^	1825.4 ± 144.63^b^
RBC, 10^12^/L	9.81 ± 0.94	10.10 ± 0.27	9.72 ± 0.74	MPV, fL	5.6 ± 0.32	5.63 ± 0.34	5.88 ± 0.34

Values were shown as mean ± SD; ^a, b^Significant difference compared to NC group (^a, b^*p* < 0.05).

WBC, white blood cell counts; Lymph, lymphocytes; Mon, monocytes; PDW, platelet; RBC, red blood cell counts; HGB, hemoglobin; HCT, hematocrit; MCV, mean corpuscular volume; MCH, mean corpuscular hemoglobin; MCHC, mean corpuscular hemoglobin concentration; RDW, red blood cell distribution width; PLT, platelet counts; MPV, mean platelet volume.

### 3.13 Complete genome information of *L. plantarum* E7

#### 3.13.1 Genomic map of *L. plantarum* E7 in chromosome

**Figure 9:**
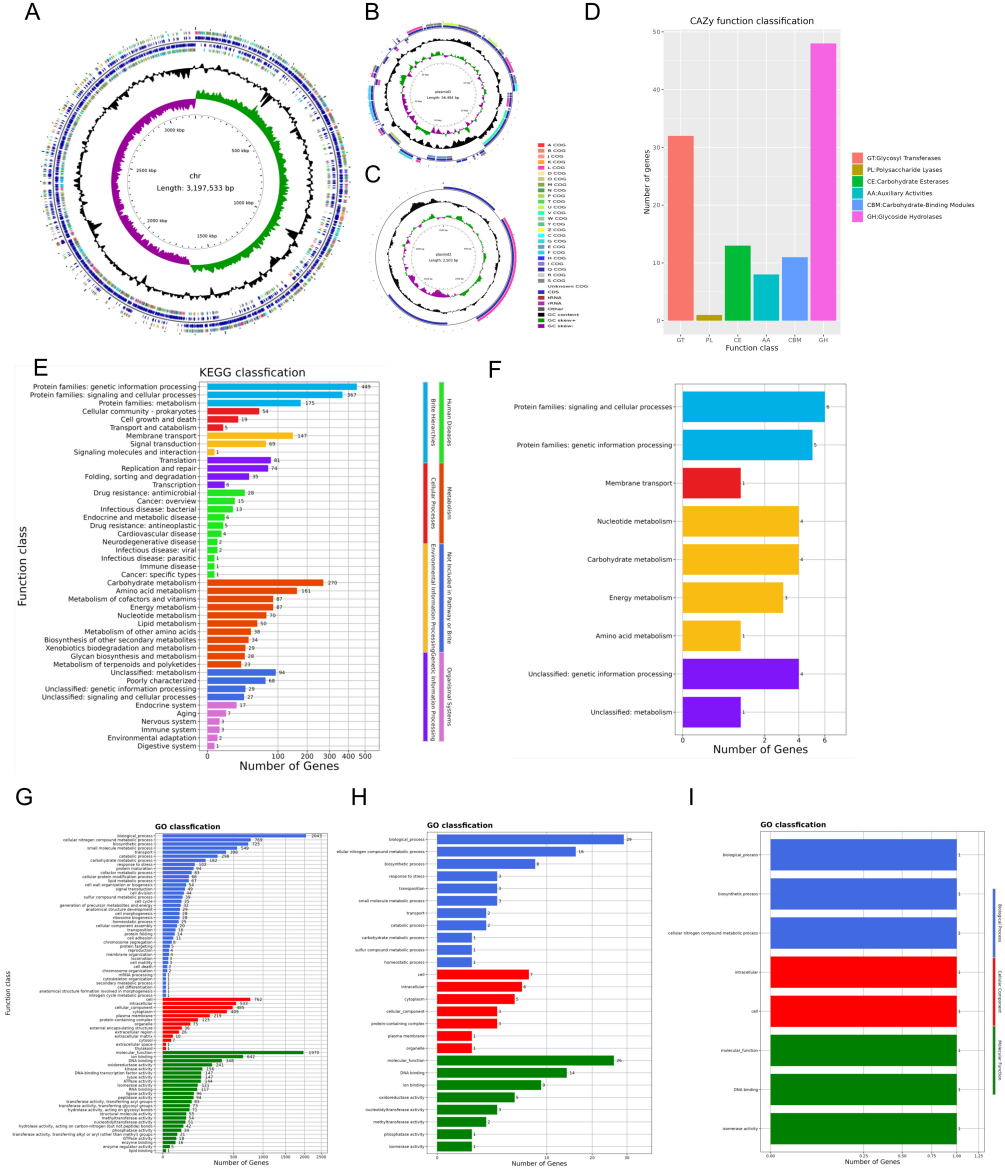
Genome analysis results of *L. plantarum* E7. **(A)** Genomic map of *L. plantarum* E7 in chromosome. **(B)** Genomic map of *L. plantarum* E7 in Plasmid 1. **(C)** Genomic map of *L. plantarum* E7 in Plasmid 2. **(D)** Functional annotation of CAZy databases of *L. plantarum* E7. **(E)** Functional annotation of KEGG databases of *L. plantarum* E7 in chromosome. **(F)** Functional annotation of KEGG databases of *L. plantarum* E7 in plasmid 1. **(G)** Functional annotation of GO databases of *L. plantarum* E7 in chromosome. **(H)** Functional annotation of GO databases of *L. plantarum* E7 in Plasmid 1. **(I)** Functional annotation of GO databases of *L. plantarum* E7 in Plasmid 2.

The *L. plantarum* E7 genome consists of a circular chromosome with a total length of 3,197,533 bp and an average GC content of 44.62 %. Two circular plasmids were identified: Plasmid 1 (56,484 bp, 39.74% GC content) and Plasmid 2 (2,503 bp, 55.97% GC content) (Supplementary Table S1). The chromosome contains 3,024 protein-coding sequences, six 5SrRNAs, five 16SrRNAs, five 23SrRNAs, 72 tRNAs, 54 ncRNAs, and one CRISPRs. The Plasmid 1 contains 58 protein-coding sequences, six ncRNAs, and no 5SrRNA, 16SrRNA, 23SrRNA, tRNA, or CRISPRs. Plasmid 2 contains three protein-coding sequences, one ncRNA, and no 5SrRNA, 16SrRNA, 23SrRNA, tRNA, or CRISPRs. From inside to outside, the diagrammatic of the genome circle shows that the first circle represents scale, the second circle represents GC Skew, the third circle represents GC-content, the fourth and seventh circles represent COG to which each CDS belongs, the fifth and sixth circles represent the positions of CDS, tRNA, and rRNA on the genome (Figures 9A–C). The complete genome sequence was uploaded to NCBI (accession number CP158575.1).

#### 3.13.2 CAZy database annotation of *L. plantarum* E7

Functional annotation of the *L. plantarum* E7 genome revealed 113 carbohydrate-active enzyme (CAZy) genes (Figure 9D), including glycosyltransferases (32 genes), polysaccharide lyases (1 gene), sugar esterases (13 genes), auxiliary activities (8 genes), and carbohydrate-binding modules (11 genes). There were 48 genes related to glycoside hydrolases (GHs). However, the plasmids had no carbohydrate active enzymes. These findings suggest that the metabolic activity of *L. plantarum* E7 focuses on carbohydrate breakdown, supporting bacterial growth, and host interaction.

#### 3.13.3 KEGG database annotation of *L. plantarum* E7

In total, 2,688 KEGG annotation genes were identified in the chromosome of *L. plantarum* E7 (Figure 9E). These genes were assigned to eight different signaling pathways. The distributing of these genes among various categories were as follows: 877 genes related to metabolism, 217 genes related to environmental information processing, 196 genes related to genetic information processing, 78 genes related to cellular processes, 78 genes related to human diseases, 991 genes related to Brite Hierarchies, 218 genes were related to Not Included in Pathway or Brite, and 33 genes related to Organismal System. Among the metabolism-related genes, the main annotations were carbohydrate metabolism (270), amino acid metabolism (164), metabolism of cofactors and vitamins (87), energy metabolism (87), lipid metabolism (50), nucleotide metabolism (70), metabolism of other amino acids (38), biosynthesis of other secondary metabolites (34), xenobiotics biodegradation and metabolism (29), metabolism of terpenoids and polyketides (23), and glycine biosynthesis and metabolism (28).

Notably, we identified critical genes for organic acid synthesis, including *alsD, budA*, and *aldC* genes encoding acetolactate decarboxylase, *ldh* gene encoding L-lactate dehydrogenase, *acKA* gene encoding acetate kinase, *pta* gene encoding phosphate acetyltransferase. This is consistent with previous findings of organic acid-mediated antimicrobial activity.

In total, 29 KEGG annotation genes were identified in the Plasmid 1 genome of *L. plantarum* E7 (Figure 9F). These genes were assigned to four different signaling pathways. The distribution of these genes among various categories were as follows: 12 genes related to metabolism, one gene related to environmental information processing, 11 genes related to Brite Hierarchies, five genes related to Not Included in Pathway or Brite. Among the metabolism-related genes, the main annotations included carbohydrate metabolism (4), amino acid metabolism (1), energy metabolism (3), and nucleotide metabolism (4). The plasmid 2 genomes had no functional annotation genes. These findings suggest that *L. plantarum* E7 has strong abilities in carbohydrates and amino acid metabolism, which could be valuable for applications in various industries such as food engineer and biologics.

#### 3.13.4 GO database annotation of *L. plantarum* E7

The chromosome of *L. plantarum* E7 was analyzed for functional prediction using the GO database, and the genic functions were divided into three categories: biological processes, cellular components, and molecular functions (Figure 9G). The majority of genes were assigned to various biological processes, including biological process (2,043), cellular nitrogen compound metabolic process (769), biosynthetic process (725), small molecule metabolic process (549), transport (398), catabolic process (298), carbohydrate metabolic process (182), and response to stress (102). In addition, *L. plantarum* E7 was found to have a variety of cellular components and molecular functions. In terms of cellular components, most of the genes were associated with cell (762), intracellular (533), cellular component (485), cytoplasm (409), plasma membrane (219), and protein containing complex (123). This suggests that *L. plantarum* E7 is involved in complex cellular components. In terms of molecular function, most genes were associated with molecular function (1970), ion binding (642), DNA binding (348), oxidoreductase activity (241), etc. These findings suggest that *L. plantarum* E7 has the ability to perform a wide range of molecular functions within its cells.

The plasmid 1 and plasmid 2 of *L. plantarum* E7 was analyzed for functional prediction using the GO database (Figures 9H, I). This also revealed similar functional categories but with reduced gene numbers.

#### 3.13.5 Potential probiotic genes of *L. plantarum* E7

Gene annotation analysis revealed that the genome of *L. plantarum* E7 contains multiple probiotic-associated genes related to acid and bile tolerance, temperature tolerance, oxidative stress, riboflavin synthesis, exopolysaccharide secretion, and cell adhesion (Supplementary Table S2). Notably, no potential probiotic genes were identified in the plasmid regions.

#### 3.13.6 Secondary metabolites of *L. plantarum* E7

AntiSMASH analysis detected four secondary metabolite biosynthesis genes clusters namely RiPP-like, T3PKS, terpene, and cyclic-lactone-autoinducer in the chromosome, with no similar gene clusters found in the plasmids (Table 7). RiPP-like and T3PK3 are two gene clusters associated with bacteriocin production. Similar gene clusters were analyzed using MiBIG database and ClusterBlast algorithm. The RiPP-like gene cluster of strain E7 and *Lactiplantibacillus plantarum* strain XJ25, *Lactiplantibacillus plantarum* strain SRCM10347 and *Lactiplantibacillus plantarum* strain LpYC41 have 100% homology. The T3PKS gene cluster has only 97% homology with *Lactiplantibacillus plantarum* strain RI-113. There are no gene clusters in the plasmids. These results indicate that *L. plantarum* E7 has the potential to produce new antibacterial substances.

**Table 7:** Secondary metabolite synthesis gene cluster of *L. plantarum* E7.

**Region**	**Type**	**From**	**To**	**Most similar known cluster**	**Similarity**
Region 1	RiPP-like	361955	374105	0	0
Region 2	T3PKS	1814322	1855491	0	0
Region 3	Terpene	2874410	2895090	0	0
Region 4	Cyclic-lactone-autoinducer	3132211	3152813	0	0

#### 3.13.7 CARD database annotation of *L. plantarum* E7

Annotation against the CARD database identified 19 antibiotic resistance-related genes (0.628% of the genome), including 11 genes associated with antibiotic targets (0.364%), one gene involved in antibiotic biosynthesis (0.033%), and 25 genes linked to overall resistance (0.827%). There were no resistance genes in the plasmids (Table 8). Results show that *L. plantarum* E7 carries antibiotic resistance genes *parC, grlB, gyrA* against fluoroquinolones, *gyrB* against aminocoumarins, *HPGAM_06235* against elfamycin, *pgsA, SAV2088* against daptomycin, and *rpoB* against rifampicin (Supplementary Table S3). This is consistent with the results of the previous antibiotic sensitivity test in which *L. plantarum* E7 was resistant to ciprofloxacin. Additionally, ResFinder analysis confirmed the absence of acquired resistance genes in the *L. plantarum* E7 genome.

**Table 8:** Antimicrobial resistance genes detected in the genome of *L. plantarum* E7.

**Property**	**Number of genes**	**Percentage (%)**
Antibiotic resistance	19	0.628
Antibiotic target	11	0.364
Antibiotic biosynthesis	1	0.033
Total genes	25	0.827

#### 3.13.8 VFDB database annotation of *L. plantarum* E7

VFDB database comparison identified a limited number of virulence genes associated with adherence, stress survival, exoenzyme activity, immune modulation, and regulation, with no virulence genes detected in the plasmids (Supplementary Table S4).

## 4 Discussion

### 4.1 Evaluation of *in vitro* probiotic properties of LAB isolated from crested ibis feces

LAB play a crucial role in maintaining gut microbiota balance and promoting intestinal health (Zeng et al., 2023). Previous studies have shown that LAB, when used as feed supplements, have a significant positive effect on animal growth. However, to ensure their safety and efficacy, it is essential to characterize and identify LAB strains. Despite the abundance of LAB in the crested ibis gut, their probiotic properties remain poorly understood. Therefore, in this study, we isolated 20 LAB strains from the feces of crested ibises and assessed their probiotic potential through a series of *in vitro* tests.

Probiotic strains must exhibit tolerance to acidic and bile salt environments to survive and function effectively in the gastrointestinal tract (Kwon et al., 2020). The gastric environment, with a pH range of 1.5–4.5 and a residence time of ~3 h, poses a significant challenge to bacterial survival. Bile salt concentrations in the small intestine range from 0.03% to 0.3% (wt/vol) (Chen et al., 2020), with food passing through the small intestine. In this study, five out of 20 isolates showed good tolerance to low pH and high bile salts conditions, consistent with findings from Akinyemi et al., who isolated 23 LAB strains from goat milk, with only six out of 23 strains showing similar tolerance (Akinyemi et al., 2024). This suggests variability in the acid and bile salt tolerance of different LAB strains. Liu et al. (2019) reported similar results with 25 strains isolated from giant panda feces, where most strains survived in bile salt conditions but did not tolerate pH 1.0. *L. plantarum* WCFS1 is one of the most intensively studied *L. plantarum* (van den Nieuwboer et al., 2016). Gastric fluid had the greatest effect on the survival of *L. plantarum* WCFS1, with a million-fold decrease in viable cells (van Bokhorst-van de Veen et al., 2012). Our study observed that four strains survived at pH 1.0, while they were still susceptible to acid environments.

Infections pathogens can cause diarrhea and inflammatory bowel disease in both humans and animals (Zhong et al., 2022). Common pathogens such as *E. coli, S. typhimurium*, and *S. aureus*, are important targets for probiotic activity (Peng et al., 2022). In this study, four LAB isolates exhibited antibacterial activity against three pathogens, excluding D8. Similar results were reported by previously where LAB isolates inhibited the growth of *E. coli* from pigs (Kaewchomphunuch et al., 2022). A previous study reported that *Lactobacillus* strains enriched with silymarin displayed significant antimicrobial activity against pathogenic bacteria and fungi (Haghshenas et al., 2023). Our study observed similar inhibitory activity, with the exception of D8.

While LAB are generally recognized as safe (GRAS), their safety must still be evaluated. In this present study, we tested the antibiotic sensitivity of five isolates. Strain E7 showed resistance to only three antibiotics, suggesting it is a safe candidate, consistent with previous findings (Coelho-Rocha et al., 2023), where most isolates were resistant to more than three antibiotics.

Surface hydrophobicity, which reflects the ability of LAB to adhere to intestinal epithelial cells, is crucial for probiotic colonization in the gut (Haghshenas et al., 2023). Among the five isolates, E7 exhibited significant medium hydrophobicity. The difference of hydrophobicity may be related to strain specificity. das Neves Selis et al. reported varying hydrophobicity in three strains of *L. plantarum* (LP03, LP289, and LP291) isolated from cocoa fermentation, with hydrophobicity levels of 36.93%, 91.67%, and 26.67%, respectively (das Neves Selis et al., 2021). In comparison, the isolates in our study showed generally weak hydrophobicity, with E7 standing out.

Hemolytic activity is a virulence factor that allows pathogens to acquire iron and other nutrients, potentially leading to anemia or edema. In our study, strain E7 exhibited no hemolytic activity, consistent with previous research that most LAB strains are non-hemolytic (Lv et al., 2022; Abdel Tawab et al., 2023).

### 4.2 *In vivo* safety evaluation of *L. plantarum* E7

*In vivo* safety assessment for selecting potential probiotics (Ren et al., 2021). Most studies suggest that strains in the range of 10^8^-10^9^ CFU/mL are often the most effective (Xu et al., 2021; Yadav et al., 2022). This study examined low-dose (4.0 × 10^8^ CFU/mL) and high-dose (4.0 × 10^10^ CFU/mL) supplementation. When the body suffers a bacterial infection, the liver and spleen become enlarged (Xu et al., 2019). High-dose *L. plantarum* E7 supplementation did not negatively affect growth performance or organ development, indicating its safety.

Hematological parameters, commonly used in histopathology, provide insights into the inflammatory response. Bacterial infections typically increase white blood cell counts, triggecircular an inflammatory response (Wanahita et al., 2003). In this study, leukocyte counts in all groups were within the normal range (0.8–6.8 × 10^9^/L), indicating that *L. plantarum* E7 does not cause inflammation. PLT increased after *L. plantarum* E7 supplementation (Versteeg et al., 2013), suggesting it may promote platelet production, consistent with previous research (Cao et al., 2024). In addition, *L. plantarum* E7 had no effect on erythrocyte-related indicators, supporting the results of the hemolytic test, which showed no toxic effect on erythrocytes.

### 4.3 Complete genome sequencing analysis provides probiotic potential for *L. plantarum* E7

Genomic analysis of *L. plantarum* E7 identified 27 genes encoding probiotic properties (Supplementary Table S2). These genes include acid tolerance (e.g., *atpA, atpB, atpC, atpD, atpE, atpF*, and *atpH*) (Ahire et al., 2021), bile salt hydrolysis (*bsh*), temperature tolerance (*groeL, csp, dnaJ*, and *dnaK*), oxidative stress defense (*arsC, trxA, trxB*, and *nfrA1*), riboflavin synthesis (*ribBA, ribE, ribF*, and *ribT*), extracellular polysaccharide synthesis (*epsF, epsH, rmlC*, and *rmlD*), and cell adhesion (*luxS, scpA*, and *scpB*). The *bsh* gene can hydrolyze bile salts and help the strains to survive in the bile salt-rich intestinal environment (Foley et al., 2023). At abnormal temperatures, proteins misfold or aggregate. Molecular chaperone genes (*groeL, csp, dnaJ*, and *dnaK*) interact with these aberrant proteins to maintain the correctly folded state of the proteins in the cell and thus play a role in thermotolerance (Gao et al., 2022). Oxidative stress-related genes (*arsC, trxA, trxB*, and *nfrA1*) help cells defend against damage caused by reactive oxygen species ROS (Wang et al., 2022). The extracellular polysaccharide synthesis gene family (*epsF, epsH, rmlC*, and *rmlD*) is involved in the synthesis, assembly and secretion of extracellular polysaccharide, and plays an important role in the biofilm formation and environmental adaptation (Vikram et al., 2022).

Good cell adhesion function is an important basis for the biological effects of LAB. Three genes related to cell adhesion (*luxS, scpA*, and *scpB*) were found in genome of *L. plantarum* E7. The discovery of these genes related to prebiotic properties is consistent with studies (Lu et al., 2024).

Annotation of the CAZy database showed that *L. plantarum* E7 encodes genes with 113 carbohydrate active enzymes. Carbohydrate active enzymes are a very important group of enzymes, including GHs, GTs, PLs, CEs, AA, and CBMs (Wardman et al., 2022). GHs are involved in the process of polysaccharide hydrolysis, which releases a lot of energy to supply various activities of bacteria. GTs use activated donors to form glycosidic bonds, transfer sugars to specific receptors such as proteins, lipids, or other glycan, and form new polymers to participate in various physiological processes. GHs and GTs were the most abundant, supporting bacterial growth on various carbon sources and playing a role in host-strain interactions and microecological balance. CEs can catalyze the de-esterification of various carbohydrate substrates. The genes encoding GHs and GTs were found to be the most numerous. This is similar to the results obtained by Chen (Chen et al., 2023). These carbohydrate enzymes contribute to the growth of *L. plantarum* E7 in a variety of carbon sources and play an important role in the interaction between strains and hosts and the maintenance of microecological balance.

Potential LAB probiotic strains are characterized for the production of inhibitory substances such as bacteriocin, hydrogen peroxide and organic acid. Key genes for organic acid synthesis were annotated in the KEGG database. This is also consistent with tests that the antimicrobial substances produced by *L. plantarum* E7 may be organic acids. Annotated results of AntiSMASH show two bacteriocin production gene clusters in *L. plantarum* E7. Aziz et al. (2024) found that there were bacteriocin gene clusters in the genome of *L. plantarum* L3. This suggests that the antimicrobial substances produced by *L. plantarum* E7 may be complex and the exact composition deserves further investigation.

Studies have shown that natural resistance in probiotics is inherent and does not transfer. However, acquired resistance has the potential to transfer (Dlamini et al., 2019). In this study, no acquired resistance genes were found in *L. plantarum* E7 and the plasmids did not contain resistance genes, suggesting a low risk of resistance transmission. We have detected the absence of genes encoding potential virulence factors such as hemolysin (*Hbl*), non-soluble enterotoxin (*Nhe*), enterohemolysin (*Cyl*), cytotoxin K (*CytK*), and other potential virulence factors, and *in vivo* and *in vitro* safety tests have also confirmed the safety of *L. plantarum* E7.

The discovery of *L. plantarum* E7 enriched the study of intestinal probiotics in crested ibis. However, the study has limitations, such as the absence of extraction and preparation of antimicrobial substances from the CFS of *L. plantarum* E7. Future studies will focus on the extraction of bacteriocins and organic acid. We will subsequently examine the lactic and acetic acid content in the CFS of *L. plantarum* E7 by mass spectrometry (LC-MS/MS). In the future, *L. plantarum E7* will be developed into a microecological preparation, which has great application potential in the study of intestinal diseases of crested ibis.

## 5 Conclusion

Taken together, the results from this study revealed that some lactic acid bacteria strains, isolated from crested ibis feces, demonstrated strong acid and bile salt resistance, pathogen inhibition activity, drug sensitivity, and hydrophobicity. Among them, *L. plantarum* E7 performed best and was also safe *in vivo* and *in vitro*. Its genome contains numerous genes linked to probiotic properties, including acid and bile salt tolerance, antimicrobial substance production, and cell adhesion. These findings provide a theoretical basis for developing new probiotics for crested ibis, with potential applications in wildlife microbiome management and disease prevention. Future research will focus on extracting antimicrobial substances and developing *L. plantarum* E7 into a microecological preparation for use in crested ibis health management.

## Data Availability

Raw sequencing data have been deposited in the NCBI Sequence Read Archive database (CP158575.1–CP158577.1).
