# Supplementary material for: Evaluation of probiotic properties and complete genome analysis of lactic acid bacteria isolated from crested ibis Nipponia nippon feces
Source: Front Microbiol. 2025 Apr 9;16:1552264. doi: 10.3389/fmicb.2025.1552264 (PMC12014687; doi:10.3389/fmicb.2025.1552264)
Supplement: Supplementary file 1 [file Supplementary_file_1.docx]

Evaluation of probiotic properties of lactic acid bacteria isolated from crested ibis *Nipponia nippon* feces

**Lei Yang^1^, Jie luo^2^, Yan Zeng^3^, Baoyue Zhang^1^, Yang Wang^1^, Gang Shu^1*^, Xiaoling Zhao^4^, Haohuan Li^1^, Funeng Xu^1^, Wei Zhang^1^, Hualin Fu^1^, Felix Kwame Amevor^4^, Rui Liu^2^**

^1^Department of Basic Veterinary Medicine, Sichuan Agricultural University, Chengdu, China 611130.

^2^Department of Animal Husbandry and Veterinary Medicine, Tongren Vocational and Technical College, Tongren, 554300.

^3^Animal Microecology Institute, College of Veterinary Medicine, Sichuan Agricultural University, Chengdu, China, 611130.

^4^Farm Animal Genetic Resources Exploration and Innovation Key Laboratory of Sichuan Province, Sichuan Agricultural University, Chengdu, Sichuan, China, 611130.

*** Correspondence:**Gang Shu
E-mail: dyysg2005@sicau.edu.c

Supplementary Material

## Supplementary Tables

**Table S1 Genome assembly and annotation of *L. plantarum* E7**

| Seq ID | Types | Numbers | Genome percentage |
| --- | --- | --- | --- |
| Chromosome | Seq Length(bp) | 3197533 bp | / |
|  | GC Content | / | 44.62 % |
|  | Total number of protein coding genes | 3024 | / |
|  | The total length of protein coding genes | 2671227 bp | 83.54 % |
|  | 5SrRNA | 6 | 0.0210% |
|  | 16SrRNA | 5 | 0.2444% |
|  | 23SrRNA | 5 | 0.4560% |
|  | tRNA | 72 | 0.1705% |
|  | ncRNA | 54 | 0.3052% |
|  | CRISPRs | 1 | / |
| Plasmid 1 | Seq Length(bp) | 56484 bp |  |
|  | GC Content | / | 39.74 % |
|  | Total number of protein coding genes | 58 | / |
|  | The total length of protein coding genes | / | 83.37 % |
|  | 5SrRNA | 0 | 0 |
|  | 16SrRNA | 0 | 0 |
|  | 23SrRNA | 0 | 0 |
|  | tRNA | 0 | 0 |
|  | ncRNA | 6 | 1.4305 % |
|  | CRISPRs | 0 | 0 |
| Plasmid 2 | Seq Length(bp) | 2503 bp | / |
|  | GC Content | / | 36.68 % |
|  | Total number of protein coding genes | 3 | / |
|  | The total length of protein coding genes | / | 55.97% |
|  | 5SrRNA | 0 | 0 |
|  | 16SrRNA | 0 | 0 |
|  | 23SrRNA | 0 | 0 |
|  | tRNA | 0 | 0 |
|  | ncRNA | 1 | 3.0364% |
|  | CRISPRs | 0 | 0 |

**Table S2 Potential probiotic genes of *L. plantarum* E7**

| Functional category | Gene symbol | Description/Function |
| --- | --- | --- |
| Acid tolerance | *atpA* | ATPase subunit alpha |
|  | *atpB* | ATPase subunit a |
|  | *atpC* | ATPase subunit epsilon |
|  | *atpD* | ATPase subunit beta |
|  | *atpE* | ATPase subunit c |
|  | *atpF* | ATPase subunit b |
|  | *atpH* | ATPase subunit delta |
| Bile Tolerance | *bsh* | bile salt hydrolase |
| oxidative stress | *arsC* | arsenate reductase |
|  | *trxA* | thioredoxin |
|  | *trxB* | thioredoxin reductase (NADPH) |
|  | *nfrA1* | FMN reductase (NADPH) |
| Riboflavin synthesis | *ribBA* | riboflavin biosynthesis RibBA |
|  | *ribE* | riboflavin synthase |
|  | *ribF* | riboflavin kinase |
|  | *ribT* | riboflavin biosynthesis RibT protein |
| Exopolysaccharide secretion | *epsF* | glycosyltransferase EpsF |
|  | *epsH* | glycosyltransferase EpsH |
|  | *rmlC* | dTDP-4-dehydrorhamnose 3,5-epimerase |
|  | *rmlD* | dTDP-4-dehydrorhamnose reductase |
| cell adhesion | *luxS* | S-ribosylhomocysteine lyase |
|  | *scpA* | segregation and condensation protein A |
|  | *scpB* | segregation and condensation protein B |
| Temperature tolerance | *gorEL* | chaperonin GroEL |
|  | *csp* | cold shock protein |
|  | *danJ* | molecular chaperone DnaJ |
|  | *dnaK* | molecular chaperone DnaK |

**Table S3 Antimicrobial resistance genes detected in the genome of *L. plantarum* E7**

| Name | Antibiotic type | Resistance  gene | Function | Source organism | Identity  (%) |
| --- | --- | --- | --- | --- | --- |
| chr_109 | Antibiotic Resistance | *mdtG* | putative drug efflux system protein | *Escherichia coli str. K-12 substr.* MG1655 | 46.05 |
| chr_1323 | Antibiotic Resistance | *arlR* | truncated (putative response regulator) | *Staphylococcus aureus subsp. aureus* Mu50 | 55.94 |
| chr_1571 | Antibiotic Resistance | *parC* | topoisomerase IV, subunit A | *Streptococcus pneumoniae* TIGR4 | 54.25 |
| chr_1572 | Antibiotic Resistance | *grlB* | DNA topoisomerase IV subunit B | *Staphylococcus aureus subsp. aureus* MRSA252 | 66.51 |
| chr_1595 | Antibiotic Resistance | *AAD01867.1* | thymidylate synthase | *Enterococcus faecalis* | 76.47 |
| chr_1795 | Antibiotic Resistance | *HPGAM_06235* | elongation factor Tu | *Helicobacter pylori Gambia* 94/24 | 73.61 |
| chr_1951 | Antibiotic Resistance | *pgsA* | Phosphatidylgly cerophosphate synthase | *Staphylococcus aureus subsp. aureus* Mu50 | 48.95 |
| chr_227 | Antibiotic Resistance | *arlR* | truncated (putative response regulator) | *Staphylococcus aureus subsp. aureus* Mu50 | 45.29 |
| chr_2558 | Antibiotic Resistance | *lmrD* | multidrug transporter | *Lactococcus lactis subsp. lactis* | 56.09 |
| chr_2559 | Antibiotic Resistance | *lmrC* | multidrug transporter | *Lactococcus lactis subsp. lactis* | 47.11 |
| chr_2680 | Antibiotic Resistance | *vanRF* | two-component response regulator | *Paenibacillus popilliae ATCC* 14706 | 50.43 |
| chr_2754 | Antibiotic Resistance | *SAV2088* | cardiolipin synthetase | *Staphylococcus aureus subsp.aureus* Mu50 | 48.75 |
| chr_2831 | Antibiotic Resistance | *mdtG* | putative drug efflux system protein | *Escherichia coli str. K-12 substr.* MG1655 | 46.71 |
| chr_30 | Antibiotic Resistance | *vanRM* | response regulator | *Enterococcus faecium* | 47.32 |
| chr_5 | Antibiotic Resistance | *gyrB* | DNA gyrase subunit B | *Staphylococcus aureus subsp. aureus* MRSA252 | 65.12 |
| chr_6 | Antibiotic Resistance | *gyrA* | DNA gyrase subunit A | *Staphylococcus aureus subsp. aureus* MRSA252 | 58.90 |
| chr_861 | Antibiotic Resistance | *rpoB* | DNA-directed RNA polymerase subunit beta | *Staphylococcus aureus subsp. aureus* MRSA252 | 70.86 |
| chr_862 | Antibiotic Resistance | *rpoC* | DNA-directed RNA polymerase subunit beta | *Staphylococcus aureus subsp. aureus* MRSA252 | 69.13 |
| chr_864 | Antibiotic Resistance | *StrA* | Unknown product | *Haemophilus influenzae* | 68.38 |
| chr_852 | Antibiotic Biosynthesis | *YBT020_04620* | acetolactate synthase | *Bacillus thuringiensis serovar finitimus* YBT-020 | 60.90 |
| chr_862 | Antibiotic Target | *rpoC* | DNA-directed RNA polymerase subunit beta prime | *Enterococcus faecium* DO | 81.25 |
| chr_861 | Antibiotic Target | *rpoB* | DNA-directed RNA polymerase subunit beta | *Enterococcus faecium* DO | 79.70 |
| chr_6 | Antibiotic Target | *gyrA* | DNA topoisomerase (ATP-hydrolyzing) subunit A | *Enterococcus faecium* DO | 73.19 |

**Table S3 Antimicrobial resistance genes detected in the genome of *L. plantarum* E7**

**(continued 1)**

| Name | Antibiotic type | Resistance gene | Function | Source organism | Identity (%) |  |
| --- | --- | --- | --- | --- | --- | --- |
| chr_421 | Antibiotic Target | *alr* | alanine racemase | *Enterococcus faecalis* V583 | 56.72 |  |
| chr_5 | Antibiotic Target | *gyrB* | DNA gyrase, B subunit | *Enterococcus faecalis* V583 | 72.42 |  |
| chr_1816 | Antibiotic Target | *SaurJH1_1191* | GTP-binding protein TypA | *Staphylococcus aureus subsp. aureus* JH1 | 68.48 |  |
| chr_1572 | | Antibiotic target | *parE* | DNA topoisomerase IV, B subunit | *Enterococcus faecalis* V583 | 77.62 |
| chr_1338 | Antibiotic Target | *BN418_1758* | Transcription elongation factor GreA | *Listeria monocytogenes* | 59.87 |  |
| chr_1364 | Antibiotic target | *rplU* | 50S ribosomal protein L21 | *Azoarcus sp.* BH72 | 48.54 |  |
| chr_1571 | Antibiotic target | *parC* | DNA topoisomerase (ATP-hydrolyzing) | *Enterococcus faecium* DO | 60.37 |  |
| chr_866 | Antibiotic Target | *fusA* | elongation factor G | *Streptococcus pneumoniae Taiwan*19F-14 | 75.21 |  |

**Table S4 Virulence factor genes of *L. plantarum* E7**

| Virulence factor classification | Gene | Function |
| --- | --- | --- |
| Adherence | *gorEL* | chaperonin GroEL |
| Immune modulation | *Hasc、galU* | UTP-glucose-1-phosphate uridylyltransferase HasC |
| Stress survival | *clpP* | ATP-dependent Clp protease proteolytic subunit |
| Exoenzyme | *eno* | phosphopyruvate hydratase |
| Immune modulation | *rfbA、rmlA、wbtL* | glucose-1-phosphate thymidylyltransferase RfbA |
| Immune modulation | *rfbB* | dTDP-glucose 4,6-dehydratase |
| Immune modulation | *cpsI、glf* | UDP-galactopyranose mutase |
| Immune modulation | *bpsD* | bacterial sugar transferase family protein |
| Immune modulation | *gndA* | NADP-dependent phosphogluconate dehydrogenase |
| Regulation | *lisR* | two-component response regulator |
| Adherence | *dnaK* | chaperone protein DnaK |
| Immune modulation | *cosA/uppS* | undecaprenyl diphosphate synthase |
| Regulation | *sigA/rpoV* | RNA polymerase sigma factor |
| Adherence | *tuf、tufA* | elongation factor Tu |
